# Supplementary material for: Transcriptome Analysis Reveals Vimentin-Induced Disruption of Cell–Cell Associations Augments Breast Cancer Cell Migration
Source: Cells. 2022 Dec 13;11(24):4035. doi: 10.3390/cells11244035 (PMC9776984; doi:10.3390/cells11244035)
Supplement: Supplementary file 1 [file cells-11-04035-s001.zip › DEGs and enrichment analysis.zip!/DEGs and enrichment analysis/WT_VIMvsVector KEGG/ALL/WT_VIMvsVector_ALL.pathway_map.zip!/WT_VIMvsVector_ALL.pathway_map/src/hsa04371.html]

hsa04371


- hsa:51764

- Up regulated genes

ENSG00000127588(3.22298284123795)

- hsa:1490

- Down regulated genes

ENSG00000118523(-2.25176450092829)

- hsa:10891

- Down regulated genes

ENSG00000109819(-2.69536333677731)

- hsa:107

- Up regulated genes

ENSG00000164742(1.87205666251591)

- hsa:51764

- Up regulated genes

ENSG00000127588(3.22298284123795)

- hsa:23710

- Down regulated genes

ENSG00000139112(-1.85791559298273)

- hsa:51764

- Up regulated genes

ENSG00000127588(3.22298284123795)

Close
